# Comparative genomics reveals genes significantly associated with woody hosts in the plant pathogen *Pseudomonas syringae*

**DOI:** 10.1111/mpp.12423

**Published:** 2016-07-15

**Authors:** Reuben W. Nowell, Bridget E. Laue, Paul M. Sharp, Sarah Green

**Affiliations:** ^1^ Institute of Evolutionary Biology, University of Edinburgh Edinburgh EH9 3FL UK; ^2^ Centre for Ecosystems, Society and Biosecurity Forest Research Midlothian EH25 9SY UK; ^3^ Centre for Immunity, Infection and Evolution University of Edinburgh Edinburgh EH9 3FL UK; ^4^ Present address: Department of Life Sciences Imperial College London Silwood Park Campus London SL5 7PY UK

**Keywords:** adaptation, genome fluctuation, *Pseudomonas syringae*, woody hosts

## Abstract

The diversification of lineages within *Pseudomonas syringae* has involved a number of adaptive shifts from herbaceous hosts onto various species of tree, resulting in the emergence of highly destructive diseases such as bacterial canker of kiwi and bleeding canker of horse chestnut. This diversification has involved a high level of gene gain and loss, and these processes are likely to play major roles in the adaptation of individual lineages onto their host plants. In order to better understand the evolution of *P. syringae* onto woody plants, we have generated *de novo* genome sequences for 26 strains from the *P. syringae* species complex that are pathogenic on a range of woody species, and have looked for statistically significant associations between gene presence and host type (i.e. woody or herbaceous) across a phylogeny of 64 strains. We have found evidence for a common set of genes associated with strains that are able to colonize woody plants, suggesting that divergent lineages have acquired similarities in genome composition that may form the genetic basis of their adaptation to woody hosts. We also describe in detail the gain, loss and rearrangement of specific loci that may be functionally important in facilitating this adaptive shift. Overall, our analyses allow for a greater understanding of how gene gain and loss may contribute to adaptation in *P. syringae*.

## Introduction

Lineages from the *Pseudomonas syringae* species complex are the causal agents of a variety of blight, speck, spot and canker diseases on a range of economically and environmentally important plant species (Hirano and Upper, 1990; Mansfield *et al*., 2012; O'Brien *et al*., 2011). The *P. syringae* species complex is divided into more than 50 pathological variants (pathovars), named for their ability to infect different plant species, which are distributed across at least seven distinct phylogenetic groups (phylogroups, PGs) based on sequence divergence of housekeeping genes (e.g. Berge *et al*., 2014; Hwang *et al*., 2005; Sarkar and Guttman, 2004). Recently, a number of pathovars have been responsible for the emergence of highly damaging new diseases of woody species, including European horse chestnut (Webber *et al*., 2008), kiwifruit (Balestra *et al*., 2010), olive (Rodríguez‐Moreno *et al*., 2009) and hazelnut (Scortichini *et al*., 2002). These epidemics have prompted a number of investigations into the genetic basis of the adaptation of *P. syringae* onto woody hosts, and the evolutionary processes that have enabled this adaptation (e.g. Green *et al*., 2010; Marcelletti *et al*., 2011; O'Brien *et al*., 2012; Rodríguez‐Palenzuela *et al*., 2010).

Genome fluctuation, defined as the gain and loss of genes through time, is an extensive evolutionary force in *P. syringae*, and previous studies have revealed the breadth and depth of the potential gene pool available via horizontal gene transfer (HGT) (e.g. Baltrus *et al*., 2011; Nowell *et al*., 2014; O'Brien *et al*., 2012). Both gene gain and loss have been implicated as important adaptive mechanisms in *P. syringae* evolution, with much focus on the repertoire dynamics of effector genes of the type III secretion system (T3SS) (e.g. Lindeberg *et al*., 2006; Ma *et al*., 2006; Pitman *et al*., 2005). The magnitude of genome fluctuation is remarkable—individual lineages may be exposed to hundreds, perhaps even thousands, of new genes within the same time frame as 1% divergence accrues among protein sequences of the core genome (Nowell *et al*., 2014). In addition, it is now known that genetically diverse populations of *P. syringae* thrive in a multitude of environmental (i.e. non‐plant) habitats, including leaf litter, river headwaters and snow‐pack (Monteil *et al*., 2012, 2013, 2014; Morris *et al*., 2009). Given this naturally occurring reservoir of genetic diversity, Monteil *et al*. (2013) have recently suggested an epidemic population structure for *P. syringae*, whereby clonal expansions of highly virulent lineages emerge from a frequently recombining and genetically diverse background population. Taken together, these findings suggest that the flexible genomes of phytopathogenic *P. syringae* lineages are adapted to be selectively advantageous when expressed in a particular niche—that of a compatible host species—and implicate HGT and gene loss as key evolutionary mechanisms that facilitate adaptation.

Here, in the light of the recent disease epidemics produced by canker‐causing pathovars, we test this hypothesis by investigating the genomic basis of *P. syringae* adaptation into an environment that has been colonized multiple times during its evolutionary history—specifically, the woody organs of a range of host species. We augment the current genomic resource for *P. syringae* with draft genomes of 26 strains (16 pathovars) that are pathogenic on a range of woody species, and delimit the *P. syringae* pan‐genome into its constituent core (genes that are shared in all taxa) and flexible (genes that occur variably) genome components. We employ these data to investigate the adaptation of *P. syringae* onto woody hosts using three different approaches.

First, we look for statistically significant correlations between flexible genes and host type among a total of 64 strains for which high‐quality, whole‐genome sequence data are available, using a method that is able to account for phylogenetic relatedness among strains. Second, we elucidate the distribution of a range of both secreted and non‐secreted virulence factors that are known to be important in *P. syringae* pathogenesis. Lastly, we reconstruct the evolutionary history of gene gain along the phylogenetic lineage leading to pathovar (pv.) *aesculi*, the causal agent of horse chestnut bleeding canker in the European horse chestnut (*Aesculus hippocastanum*), and assess the putative functions of acquired genes in relation to their potential role in pathogenesis.

## Results

### Genome sequencing and assembly

We selected 26 strains of *P. syringae* (16 pathovars) that are pathogens of a wide range of woody plants for whole‐genome sequencing using Illumina MiSeq technology (Table 1). The resultant draft assemblies ranged in span from 5.62 to 6.47 Mb, with a median of 6.19 Mb (Table S1, see Supporting Information). Assembly N50, defined as the length of the contig at which 50% of the genome is covered by a contig of equivalent length or longer, ranged from 41.8 to 246.4 kb (median of 66.3 kb), and all genomes were assembled into fewer than 400 contigs. Overall, data retention during assembly was high in all cases, with ≥97% of filtered reads aligning to the final assembly for each strain. Gene repertoire ‘completeness’ was also high, with only one core protein (from a total of 40; Simão *et al*., 2015) absent from each assembly.

**Table 1:** Strain information.

Pathovar	Strain	Identifier*	Host	Year^†^	Contigs	CDS^‡^	Trait^§^	Reference
*actinidiae*	MAFF 302091	*actn302091*	*Actinidia deliciosa* (kiwifruit)	1984	941	5169	W	Baltrus *et al*. ( 2011)
*actinidiae*	NCPPB 3739	*actn3739*	*Actinidia deliciosa* (kiwifruit)	1984	815	5283	W	Marcelletti *et al*. ( 2011)
*actinidiae*	NCPPB 3871	*actn3871*	*Actinidia deliciosa* (kiwifruit)	1992	466	5267	W	Marcelletti *et al*. ( 2011)
*actinidiae*	CRAFRU8.43	*actn843*	*Actinidia deliciosa* (kiwifruit)	2008	585	5513	W	Marcelletti *et al*. ( 2011)
*aesculi*	NRS 2113	*aesc2113*	*Aesculus hippocastanum* (European horse chestnut)	2006	330	5644	W	This study
*aesculi*	NRS 2250	*aesc2250*	*Aesculus hippocastanum* (European horse chestnut)	2008	776	5324	W	Green *et al*. ( 2010)
*aesculi*	NRS 2279	*aesc2279*	*Aesculus hippocastanum* (European horse chestnut)	2002	322	5688	W	This study
*aesculi*	NRS 2306	*aesc2306*	*Aesculus hippocastanum* (European horse chestnut)	2010	291	5734	W	This study
*aesculi*	NRS 2315	*aesc2315*	*Aesculus hippocastanum* (European horse chestnut)	2006	289	5623	W	This study
*aesculi*	NRS 2329	*aesc2329*	*Aesculus hippocastanum* (European horse chestnut)	2011	319	5797	W	This study
*aesculi*	NRS 2336	*aesc2336*	*Aesculus hippocastanum* (European horse chestnut)	2010	288	5717	W	This study
*aesculi*	NRS 3681	*aesc3681*	*Aesculus indica* (Indian horse chestnut)	1979	841	5293	W	Green *et al*. ( 2010)
*alisalensis* ^¶^	ES4326	*Pcan4326*	*Raphanus sativus* (radish)	1965	878	5475	H	Baltrus *et al*. ( 2011)
*aptata*	DSM 50252	*apta50252*	*Beta vulgaris* (sugar beet)	1948	3776	5265	H	Baltrus *et al*. ( 2011)
*atrofaciens*	DSM 50255	*atro50255*	*Triticum aestivum* (wheat)	1974	669	5040	H	Baltrus *et al*. ( 2014a)
*atrofaciens*	LMG 5095	*atro5095*	*Triticum aestivum* (wheat)	1974	1007	5160	H	Y.‐H. Noh and J.‐S. Cha (unpublished data)
*avellanae*	ISPaVe037	*avel037*	*Corylus avellana* (hazel)	1992	317	5321	W	O'Brien *et al*. ( 2012)
*avellanae*	ISPaVe013	*avel013*	*Corylus avellana* (hazel)	1992	191	5172	W	O'Brien *et al*. ( 2012)
*avellanae*	BPIC631	*avel631*	*Corylus avellana* (hazel)	1976	1602	5228	W	O'Brien *et al*. ( 2012)
*avellanae*	CRAFRUec1	*avelec1*	*Corylus avellana* (hazel)	2003	547	5160	W	Scortichini *et al*. ( 2013)
*avii*	CFBP 3846	*avii3846*	*Prunus avium* (cherry)	1991	324	5680	W	This study
—	BRIP 34876	*BRIP34876*	*Hordeum vulgare* (barley)	1971	148	5119	H	Gardiner *et al*. ( 2013)
—	BRIP 34881	*BRIP34881*	*Hordeum vulgare* (barley)	1971	157	5136	H	Gardiner *et al*. ( 2013)
—	BRIP 39023	*BRIP39023*	*Hordeum vulgare* (barley)	1988	34	5123	H	Gardiner *et al*. ( 2013)
*broussonetiae*	CFBP 5140	*brou5140*	*Broussonetia kazinoki* (paper mulberry)	1980	359	5784	W	This study
*castaneae*	CFBP 4217	*cast4217*	*Castanea crenata* (Japanese chestnut)	1977	220	5710	W	This study
*cerasicola*	CFBP 6109	*cera6109*	*Prunus yedoensis* (Yoshino cherry)	1995	353	5415	W	This study
—	Cit7	*cit7*	*Citrus sinensis* (navel orange)	2008	2655	5321	H	Baltrus *et al*. ( 2011)
*daphniphylli*	CFBP 4219	*daph4219*	*Daphniphyllum teijsmanni*	1981	370	5697	W	This study
*dendropanacis*	CFBP 3226	*dend3226*	*Dendropanax trifidus* (ivy tree)	1979	219	5334	W	This study
*eriobotryae*	CFBP 2343	*erio2343*	*Eriobotrya japonica* (loquat tree)	1970	129	5733	W	This study
*fraxini*	CFBP 5062	*frax5062*	*Fraxinus excelsior* (ash tree)	1978	331	5723	W	This study
*glycinea*	B076	*glycB076*	*Glycine max* (soybean)	2007	104	5613	H	Qi *et al*. ( 2011)
*glycinea*	race 4	*glycR4*	*Glycine max* (soybean)	1977	108	5314	H	Qi *et al*. ( 2011)
*japonica*	MAFF 301072	*japo301072*	*Hordeum vulgare* (barley)	1951	4,661	5562	H	Baltrus *et al*. ( 2011)
*lachrymans*	MAFF 301315	*lach301315*	*Cucumis sativus* (cucumber)	1975	791	6275	H	Baltrus *et al*. ( 2011)
*lachrymans*	MAFF 302278	*lach302278*	*Cucumis sativus* (cucumber)	1935	798	5265	H	Baltrus *et al*. ( 2011)
*morsprunorum*	NRS 2341	*mors2341*	*Prunus cerasus* (wild cherry)	1988	173	5692	W	This study
*morsprunorum*	MAFF 302280	*mors302280*	*Prunus domesticus* (European plum)	1977	969	5338	H**	Baltrus *et al*. ( 2011)
*morsprunorum*	HRI‐W 5261	*mors5261*	*Prunus avium* (sweet cherry cv. Roundel)	1990	264	5887	W	This study
*morsprunorum*	HRI‐W 5269	*mors5269*	*Prunus cerasus* (sour cherry)	1990	158	5580	W	This study
*myricae*	CFBP 2897	*myri2897*	*Myrica rubra* (Chinese bayberry)	1978	204	5421	W	This study
*nerii*	CFBP 5067	*neri5067*	*Nerium oleander* (oleander)	1979	242	5249	W	This study
*panici*	LMG 2367	*pani2367*	*Panicium miliaceum* (proso millet)	1963	148	5154	H	Liu *et al*. ( 2012)
*papulans*	CFBP 1754	*papu1754*	*Malus sylvestris* (crab apple)	1973	174	5705	W	This study
*phasiolicola*	1448A	*phas1448A*	*Phaseolus vulgaris* (common bean)	1985	3	5172	H	Joardar *et al*. ( 2005)
*pisi*	PP1	*pisiPP1*	*Pisum sativum* (pea)	1978	256	5157	H	Baltrus *et al*. ( 2014b)
*rhaphiolepidis*	CFBP 4220	*rhap4220*	*Rhaphiolepis umbellata* (yeddo hawthorn)	1980	292	5159	W	This study
*savastanoi*	NCPPB 3335	*sava3335*	*Olea europaea* (olive tree)	1984	403	5194	W	Rodríguez‐Palenzuela *et al*. ( 2010)
*syringae*	1212	*syri1212*	*Pisum sativum* (pea)	—	338	5324	H	Baltrus *et al*. ( 2014b)
*syringae*	NRS 2339	*syri2339*	*Prunus avium* (sweet cherry)	1984	69	5246	W	This study
*syringae*	NRS 2340	*syri2340*	*Pyrus* sp. (pear)	1985	98	5354	W	This study
*syringae*	642	*syri642*	Not stated	2007	296	5100	H	Clarke *et al*. ( 2010)
*syringae*	HRI‐W 7872	*syri7872*	*Prunus domestica* (plum cv. Opal)	2000	105	5058	W	This study
*syringae*	HRI‐W 7924	*syri7924*	*Prunus cerasus* (sour cherry)	2000	130	5478	W	This study
*syringae*	B301D‐R	*syriB301*	*Pyrus communis* (pear flower)	1969	81	5168	H	Dudnik and Dudler ( 2014)
*syringae*	B728a	*syriB728a*	*Phaseolus vulgaris* (common bean)	1987	1	5089	H	Feil *et al*. ( 2005)
*tabaci*	ATCC 11528	*taba11528*	*Nicotiana tabacum* (tobacco)	1905	1405	5432	H	Studholme *et al*. ( 2009)
*tabaci*	6605	*taba6605*	*Nicotiana tabacum* (tobacco)	1967	284	5441	H	D. J. Studholme *et al*. (unpublished data)
*theae*	ICMP 3923	*thea3923*	*Camellia sinensis* (tea plant)	1974	378	5633	W	Mazzaglia *et al*. ( 2012)
*tomato*	NCPPB 1108	*toma1108*	*Solanum lycopersicum* (tomato)	1961	304	5467	H	Cai *et al*. ( 2011)
*tomato*	DC3000	*tomaDC3000*	*Solanum lycopersicum* (tomato)	1960	3	5619	H	Buell *et al*. ( 2003)
*tomato*	T1	*tomaT1*	*Solanum lycopersicum* (tomato)	1986	122	5583	H	Almeida *et al*. ( 2009)
*ulmi*	CFBP 1407	*ulmi1407*	*Ulmus* sp. (elm)	1958	323	5933	W	This study

*Unique identifier used in this study.

^†^Year of original isolation (if known).

^‡^Number of coding sequences (CDS) as annotated by Rapid Annotation using Subsystem Technology (RAST).

^§^Trait designation based on host type: H, herbaceous host; W, woody host (see Experimental Procedures).

^¶^Originally identified as *P. syringae* pv. *maculicola*, this strain has been reclassified recently as *Pseudomonas cannabina* pv. *alisalensis* (Bull *et al*., 2010).

**As mentioned by Gardan *et al*. (1999) and Ménard *et al*. (2003). See Table S5 in Supporting Information for source abbreviations.

These data were combined with 38 publicly available genome sequences from across the *P. syringae* species complex. Reannotation of these 64 strains produced a total of 348 022 protein‐coding genes, the products of which were then clustered into 11 200 initial groups by OrthoMCL. After applying the correction procedures outlined in Nowell *et al*. (2014), the size of the core genome was estimated at 2677 genes, or ∼48% of the total number of genes in an average *P. syringae* genome. The pan‐genome was estimated at 13 010 genes (Fig. S1, see Supporting Information).

### Phylogenetics

**Figure 1:**
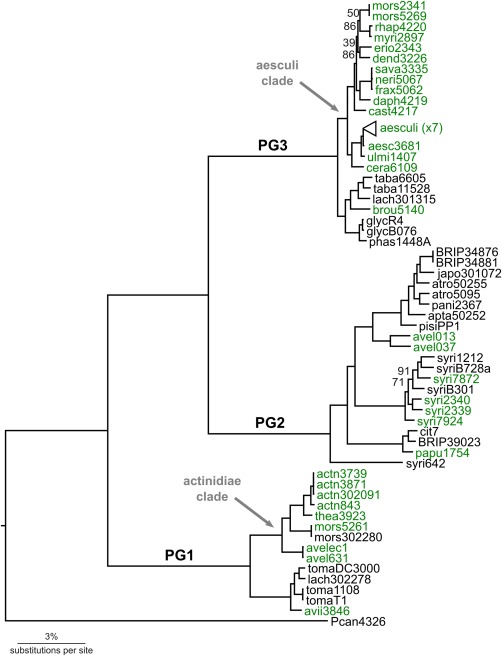
Maximum likelihood phylogeny of 64 strains from the *Pseudomonas syringae* species complex. All nodes have at least 98% bootstrap support, except where indicated. Taxon names in green are strains isolated from woody hosts. Major phylogroups (PGs) 1, 2 and 3 are shown on the branches; the two major clades of woody host pathogens are also indicated. The tree is rooted with *Pseudomonas cannabina* pv. *alisalensis* str. ES4326 (*Pcan4326*); scale bar indicates 0.03 substitutions per site.

The core genome phylogeny was reconstructed from the 1.15 Mb concatenated nucleotide alignment of 2086 one‐to‐one orthologous genes using maximum likelihood (Fig. 1). This shows the well‐supported partitioning of these strains into three clusters, corresponding to PGs 1, 2 and 3, as defined by Sarkar and Guttman (2004). Strains inferred to be pathogens of woody hosts, indicated in green on the phylogeny, fall within each of the three main PGs and are not monophyletic within any PG. The majority of woody host strains (∼75%) cluster within two clades. The largest is in PG3, and contains all of the PG3 woody host strains with the exception of pv. *broussonetiae*; this is designated as the ‘aesculi’ clade. The other is found in PG1 and is designated as the ‘actinidiae’ clade.

### Correlated evolution between gene presence and woody hosts

We used the program BayesTraits (Pagel, 1994) to look for statistically significant correlations between gene presence and the ability to colonize the woody parts of a host plant (the ‘woody niche’) by way of a likelihood ratio (LR) test. The shape of the observed LR distribution suggests an excess of genes with an LR value greater than the threshold indicated by the null (Fig. S2, see Supporting Information). The numbers of genes exceeding each threshold are shown in Table 2, together with the expected number of Type I (false‐positive) errors under the null model. Of the 3883 tested sites of the flexible genome, 899 have an LR value that exceeds the *P* ≤ 0.05 threshold. The expected number of false positives is 194, implying that there are about 700 genes (i.e. ∼18% of tested genes or ∼7% of all flexible genes) showing a significant association with strains that colonize the woody parts of their host.

**Table 2:** Number of genes significantly associated with the woody niche.

*P* value	LR value	Number of genes		Proportion (%)	
		Expected*	Observed	Tested^†^	Flexible^‡^
0.05	6.78	194	899	18.15	6.82
0.01	9.50	39	296	6.62	2.49
0.001	13.02	4	59	1.42	0.53
0.0001	16.50	<1	20	0.51	0.19
0.00001	20.89	≪1	3	0.08	0.03

*Expected number of Type I (false‐positive) errors under the null model.

^†^Proportion of the 3883 tested genes.

^‡^Proportion of the total flexible genome (10 333 genes).

**Figure 2:**
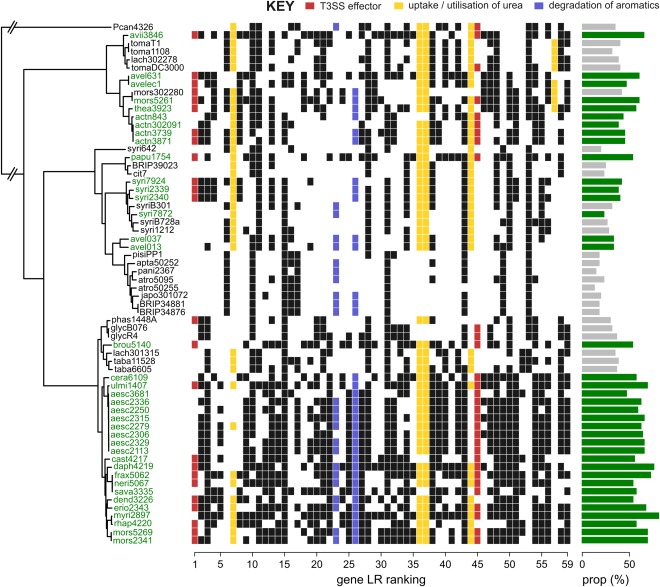
Occurrence profile for 59 genes significantly associated with the woody niche. Genes of particular interest are highlighted in colour (see key). Genes are ordered from 1 to 59 corresponding to the magnitude of the likelihood ratio (LR) statistic (decreasing significance); the order of genes is not indicative of physical proximity on the chromosome. Strains are ordered according to the core genome phylogeny; the bar chart on the right shows the proportion of genes (out of 59) present in woody (green) versus non‐woody (grey) host pathogens. T3SS, type III secretion system.

To gain a better understanding of the nature of this association, we plotted the patterns of occurrence of the 59 genes associated with the woody niche at *P* ≤ 0.001 (Fig. 2). Most of these genes (47 of 59) are not found exclusively in woody host strains, but are present in multiple transitions from herbaceous to woody hosts in the phylogeny. On average, woody host strains possess 33 of the 59 genes (56%), compared with about 18 (30%) in non‐woody strains.

The putative functions of these genes were ascertained using evidence from gene orthology. Twenty genes (∼34%), including five of the top 10, were either annotated as hypothetical proteins or returned no matches. A further 10 genes (∼17%) were described as having functions related to either transposition or conjugal transfer. The putative functions for the remaining 29 genes are shown in Table S2 (see Supporting Information). Two proteins show sequence identity to known type III secretion effector proteins (HopAY1 and HopAO1), whereas six proteins are involved in the uptake, transport or utilization of urea. In addition, 4‐oxalocrotonate tautomerase (gene #23) and muconate cycloisomerase (gene #26) both have roles in the degradation of a number of aromatic compounds, including benzene, toluene and xylene, which are constituents of extracts from wood, such as pine tar.

Physical linkage among these 59 genes was also assessed, using the *myri2897* genome as a reference, as this strain encoded the most ‘woody niche’ genes. Of the 56 genes present in *myri2897*, 32 (∼57%) hit to different contigs, and the only operon of note included five of the six genes involved in urea metabolism. Querying these genes against a database of putatively plasmid‐derived contigs (Table S3, see Supporting Information) suggests that at least 22 genes (37%) are likely to be encoded on contigs with identity to known plasmids.

### Distribution of T3SS effectors (T3SEs) and virulence genes across the *P. syringae* complex

**Figure 3:**
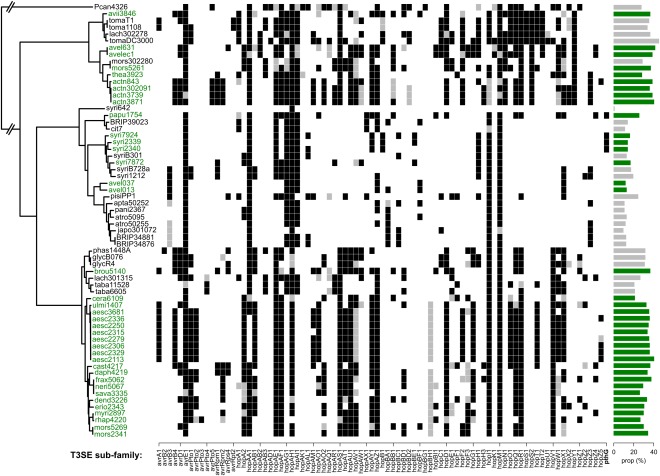
Distribution of type III secretion system effectors (T3SEs) across the *Pseudomonas syringae* species complex. Black boxes indicate presence; grey boxes indicate possible truncation. It should be noted that *avrB* is listed as present by similarity, but is known not to translocate (Baltrus *et al*., 2011). T3SE names are given at the bottom—genes designated with the same letter are within the same family, numbers indicate subfamilies. The effector with similarity to PthG from *Pantoea*, indicated in bold, is putatively from outside the *P. syringae* species complex.

We also elucidated the distribution of specific genes with known functions in *P. syringae* pathogenicity, including T3SEs and other virulence factors. The occurrence profile for 88 T3SE subfamilies is given in Fig. 3. Overall, T3SE occurrence is highly variable and does not correspond to the phylogeny of these strains. It should be noted that strain *syri642* is known to lack the canonical T3SS apparatus (Clarke *et al*., 2010).

Discounting *syri642*, repertoire size ranged from 10 (*atro5095*, *japo301072* and *pani2367*) to 41 (*tomaDC3000*). In agreement with previous analyses (e.g. Baltrus *et al*., 2011; Bartoli *et al*., 2015), strains within PG2 have many fewer T3SEs than the other two PGs (13 on average, compared with 35 and 29 for PG1 and PG3, respectively). A total of seven T3SEs (AvrPto3, HopBE1, HopBI1, HopBH1, HopH3, HopZ5 and PthG) was encoded exclusively by woody host strains in this analysis, although both HopBH1 and HopBI1 are found in the more diverged (PG4) rice pathogen pv. *oryzae* str. 1_6 (Mucyn *et al*., 2014). The average number of effectors encoded by woody host strains is 29, compared with 20 encoded by non‐woody host strains, although the phylogenetic non‐independence of these data makes the significance of this difference difficult to ascertain.

A 488‐residue protein with 92% amino acid identity to an effector encoded by the gall‐forming plant pathogen *Pantoea agglomerans* pv. *gypsophilae*, denoted PthG (Ezra *et al*., 2004), was found exclusively in the PG2 strains *syri2339*, *syri2340*, *syri7924* and *papu1754*, and has no identity to any T3SEs already described for *P. syringae*. It should be noted that the ability of this putative novel effector to be translocated (i.e. injected into a host cell via the T3SS) is not known.

**Figure 4:**
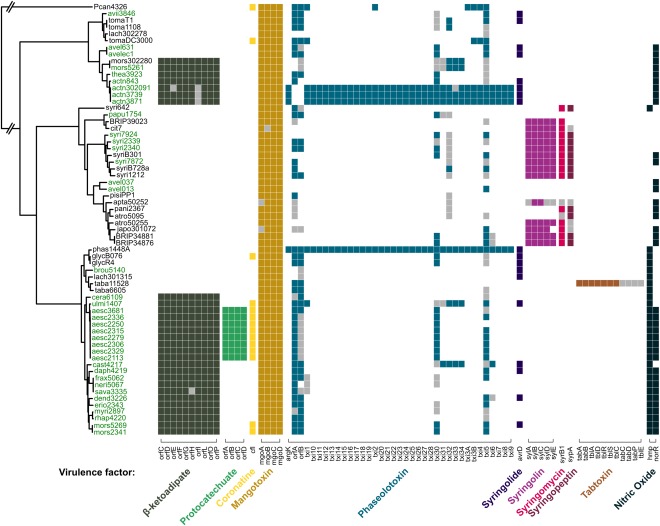
Distribution of known and suggested virulence genes across the *Pseudomonas syringae* species complex. Genes within operons are arranged into coloured blocks; grey boxes indicate the presence of a partial hit (80% identity over <80% query length) for that gene.

We also characterized the pattern of occurrence for a number of other virulence factors (Fig. 4). In agreement with previous studies (e.g. Baltrus *et al*., 2011; Hwang *et al*., 2005), patterns of occurrence are simpler than those shown by T3SEs and largely correspond to phylogeny. The β‐ketoadipate and protocatechuate‐4,5‐deoxygenase operons have been suggested previously to be potentially important adaptations of *P. syringae* to the woody niche (e.g. Bartoli *et al*., 2015; Green *et al*., 2010); thus, we focus on the distribution of these genes here. In agreement with Bartoli *et al*. (2015), the β‐ketoadipate operon is restricted to strains within PG1 and PG3. Expanding on their result, we show that this operon is present in the monophyletic ‘aesculi’ clade in PG3, and delimits host type (woody versus non‐woody) within PG3, with the exception of pv. *broussonetiae*. The operon is also present in pathovars *actinidiae*, *theae* and *morsprunorum* within the PG1 ‘actinidiae’ clade, but is not found in the closely related hazelnut pathogens from the pathovar *avellanae* (strains *avel631* and *avelec1*). In contrast, the protocatechuate‐4,5‐deoxygenase pathway was found to be unique to pv. *aesculi*.

### Genomic adaptations to the woody niche along the *aesculi* lineage

**Figure 5:**
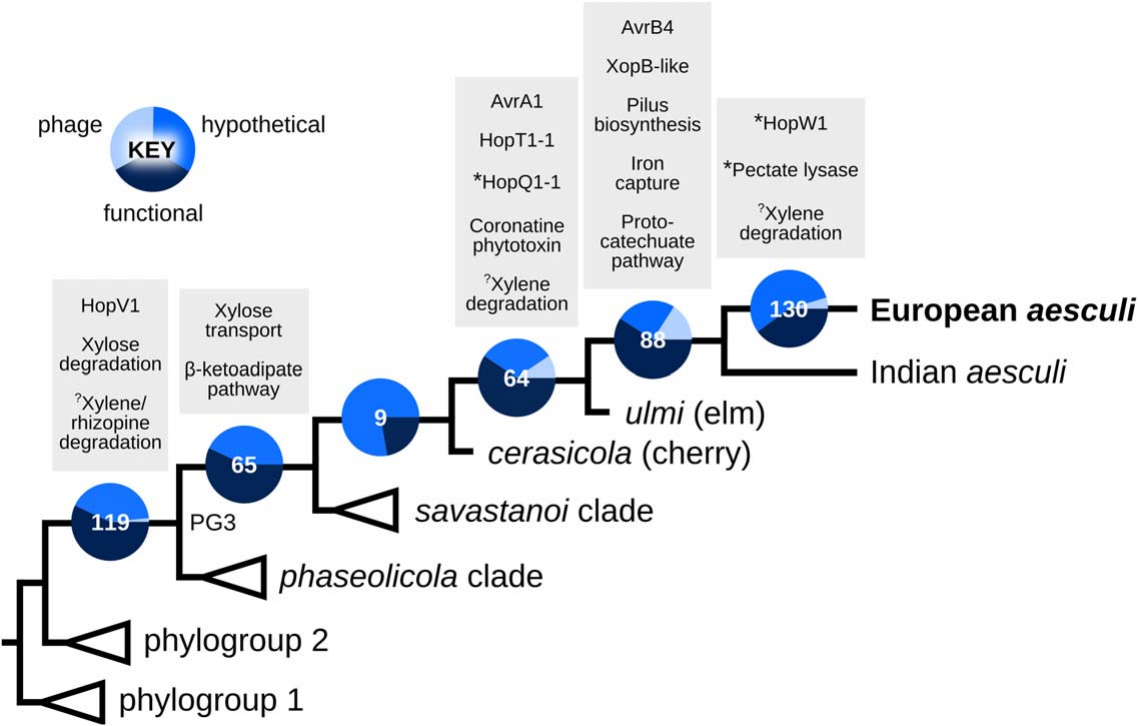
Gene gain along the phylogenetic lineage leading to *Pseudomonas syringae* pv. *aesculi*. The number of well‐supported gene gains is indicated for each branch, delimited into three basic categories (see key). Genes/functions of specific interest with respect to the adaptation of *P. syringae* into the woody niche are listed above each branch. Asterisks denote partial or truncated genes; question marks denote an incomplete pathway or where the gain of function is unclear. Topology is based on the core genome phylogeny (branch lengths not to scale).

In order to gain a clearer understanding of the evolution of *P. syringae* into the woody niche, we investigated the history of gene gain along the phylogenetic lineage leading to pv. *aesculi* (Fig. 5; see also Dataset S1 in Supporting Information). This reveals a number of potentially important adaptations to the woody niche, outlined below.

Our reconstruction shows the gain of a gene encoding a 278‐amino‐acid protein annotated as a putative xylose isomerase, involved in the utilization of the wood‐derived sugar d‐xylose, at the root of all PG3 pathovars. Mapping of this gene to the *aesc2336* assembly showed it to be independent of the alternative xylose degradation operon (*xylRAFGH*) which is ubiquitous across the *P. syringae* species complex. This operon also contains a xylose isomerase gene, that we denote *xylA*_1_, but these two genes are not similar—the PG3 xylose isomerase (denoted *xylA*_2_) is 160 codons shorter than *xylA*_1_, and alignment of the two reveals very low amino acid identity (∼15%). The *xylA*_2_ gene is present in all PG3 strains, but also in the relatively distantly related pathovars *actinidiae* and *theae* in PG1.

Phylogenetic analysis of *xylA*_2_ revealed that, although PG1 and PG3 homologues were clearly partitioned, the level of divergence across all sites (*p* distance) was much reduced relative to that of *xylA*_1_ (0.07 versus 0.25). Further investigation revealed this difference to be primarily driven by divergence at synonymous sites (*K*_s_), with values of 0.63 and 0.12 for *xylA*_1_ and *xylA*_2_, respectively (Table S4, see Supporting Information). In addition, two further genes with putative functions in the transport of d‐xylose across the cell membrane were inferred to have been acquired at the root of the ‘aesculi’ clade in PG3. As was the case for *xylA*_2_, these two genes are independent of the *xylRAFGH* locus and are not similar to any component of this operon. Neither of the two genes was found outside the ‘aesculi’ clade, and they also occurred variably within this group.

In both PG1 and PG3 strains, the *xylA*_2_ gene occurs immediately downstream of three genes with putative functions in the degradation of rhizopines, compounds which are synthesized by nitrogen‐fixing bacteria within the root nodules of leguminous plants (Bahar *et al*., 1998; Murphy *et al*., 1995; Saint *et al*., 1993). This cluster of genes, denoted *mocDEF*, was also inferred to have been acquired at the root of PG3, and is similarly exclusive to PG3 strains and to pathovars *theae* and *actinidiae* in PG1.

We also found evidence for the gain of at least six T3SEs along the lineage leading to pv. *aesculi*. Of particular interest is the effector gene *hopV1*, gained along the branch ancestral to PG3. blast analysis revealed that this gene was ubiquitous among PG3 strains, but it was also found in pathovars *theae* and *tomato* str. DC3000 in PG1. Phylogenetic analysis of *hopV1* showed that the pv. *theae* homologue clustered within the PG3 clade, suggesting the recent transfer of this gene from a PG3 lineage into the pv. *theae* genome (Fig. S3, see Supporting Information). Alignment of *hopV1* to the *aesc2336* assembly showed that it was inserted immediately downstream of the *xylRAFGH* operon discussed above. Furthermore, we detected a topological discordance relative to the core genome phylogeny at the nearby *xylH* locus, such that PG1 and PG3 homologues cluster monophyletically, with PG2 basal to this group (Fig. S4, see Supporting Information), suggesting that the transfer of *hopV1* between PGs may have involved homologous recombination of the *xylH* locus.

**Figure 6:**
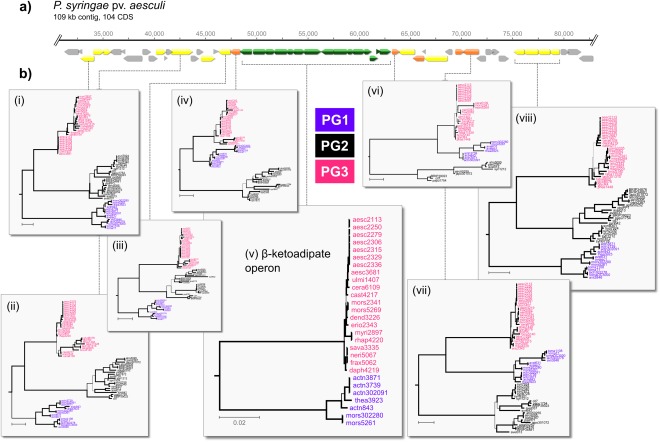
Phylogenetic history of the β‐ketoadipate operon. (a) Part of the ∼109‐kb contig from the assembly of *aesc2336* containing the β‐ketoadipate gene cluster (green). Genes in yellow have a phylogenetic history that is congruent with that of the core genome phylogeny; genes in orange show phylogenetic discordance. Grey indicates genes for which phylogenies were not estimated. (b) Selected gene phylogenies. Strains from the three phylogroups are shown in purple, black and pink for PG1, PG2 and PG3, respectively. All phylogenies are rooted with the outgroup strain *Pcan4326* (not shown), except for trees (v) and (vi) which were midpoint rooted. Branch thicknesses are drawn relative to the bootstrap support (thicker indicates higher support; no minimum bootstrap threshold). All scale bars represent 0.02 nucleotide substitutions per site.

Our reconstruction showed that the β‐ketoadipate operon had been gained at the root of the ‘aesculi’ clade in PG3. Phylogenetic analysis of the ∼7.5‐kb concatenated alignment of the 10 genes of this operon showed the well‐supported partitioning of these homologues into clusters that correspond to PGs 1 and 3 of the core genome phylogeny (Fig. 6). The observed level of divergence between PG1 and PG3 homologues, however, was approximately half that of genes of the core genome (average *p* distance of 0.127 versus 0.215). Partitioning this divergence into its constituent synonymous and non‐synonymous components showed an average *K*_s_ of 0.097 and an average *K*_a_ of 0.007, both of which are at least an order of magnitude lower than those observed for core genes (Table S4). Phylogenies of genes immediately upstream [tree (iv)] and downstream [trees (vi) and (vii)] of the operon show the clustering of PG1 with PG3, whereas phylogenies for loci further away [trees (i), (ii), (iii) and (viii)] resemble the core genome phylogeny (Fig. 6b).

## Discussion

Our analyses demonstrate a novel approach for the detection of genes that may be important in the expression of certain phenotypes by bacterial lineages. We used Pagel's (1994) method of detecting correlated evolution of discrete traits along a phylogeny, defining one trait as gene occurrence (presence or absence) and the other as the ability (or otherwise) to cause disease in the woody parts of a host plant. Below, we discuss the wider implications of our results in the context of recent literature regarding *P. syringae* population genomics and evolution, and highlight a number of genes and pathways that merit further investigation with regard to the genetic basis of *P. syringae* pathogenesis in the woody parts of host plants.

### The distribution of flexible genes contains an ecological signal that is dependent on niche type

We have found that a substantial proportion of the *P. syringae* flexible genome (∼7%, or about 700 genes) is significantly associated with the ability to colonize the woody parts of a plant host. This suggests that, for a certain fraction of the flexible genome at least, patterns of gain and loss are neither random nor strictly inherited (i.e. congruent with phylogeny); rather, they follow associations based on the ecological characteristics of these lineages—namely, the ability or otherwise to exist in the woody niche. This implies, perhaps unsurprisingly, that strains inhabiting a given ecological niche require the same, or similar, sets of functions that are encoded by the same, or similar, sets of genes, in order to proliferate. Given the extent of HGT‐mediated genome fluctuation in *P. syringae* genomes, this suggests a convergent ‘tailoring’ of the flexible genome that is determined within the ecological context of the environment in which it resides.

Our observations fit well with models regarding the role of HGT in bacterial niche adaptation (Ochman *et al*., 2005; Polz *et al*., 2013), and lend support to recent suggestions of an epidemic population structure for *P. syringae*, whereby clonal expansions of plant‐pathogenic lineages emerge from a highly diverse and recombinogenic background population that lives primarily in environmental habitats (Monteil *et al*., 2013; Vinatzer and Monteil, 2014). Although the majority of strains included in this study are plant pathogens, the results presented here suggest that HGT‐mediated genome fluctuation may also facilitate the transition of a *P. syringae* lineage from an epiphyte/environmental bacterium to a pathogen.

It follows that genes that are significantly associated with the woody niche are likely to confer a selective advantage when expressed in that environment. We note that a number of genes involved in the utilization of urea are among the set most significantly associated with the woody niche. Although these genes were not exclusive to woody host strains, we speculate that the ability to breakdown urea may be an important trait of strains that have invaded the nitrogen‐limited woody parts of host plants (Eriksson *et al*., 2012; Higuchi, 2012), although further work is needed to confirm this hypothesis. In addition, two enzymes (muconate cycloisomerase and 4‐oxalocrotonate tautomerase) have roles in the degradation of wood‐derived compounds, such as xylene and toluene. We also found that two T3SEs, HopAY1 and HopAO1, are significantly associated with the woody niche, whereas a further two (HopH3 and HopZ5) have been independently acquired by multiple woody host lineages, and are found only in strains that are pathogens of woody hosts.

It is interesting to note the large number of proteins that we infer to be either hypothetical proteins or involved in transposition among the most significantly associated genes. This may be a result of the HGT process itself, which is likely to involve mobile elements, such as plasmids and pathogenicity islands, which are rich in both insertion sequences and coding sequences of unknown function. Nonetheless, we observe a clear signal of association from these data at the genome‐wide level: when these strains are defined by the fairly broad ecological distinction of woody versus non‐woody host type, the occurrence profile of specific genes is dictated not by phylogeny, but by ecology. Thus, we suggest that these genes and pathways merit further investigation with regard to the genetic basis of *P. syringae* adaptation onto woody hosts.

### Gain, loss and rearrangement within the d‐xylose operon

Our results implicate the utilization of d‐xylose as a potentially important adaptation in woody host‐infecting pathovars in PG1 and PG3. d‐Xylose is an environmentally abundant pentose sugar, and is the primary constituent of hemicellulose xylan, itself a major component of both hard‐ and softwoods (Jeffries, 1983). We infer the gain of a number of genes involved in both the transport and isomerization of d‐xylose along lineages within both PG1 and PG3. For example, the reduced level of divergence observed for an alternative xylose isomerase gene (*xylA*_2_), involved in the incorporation of d‐xylose into the pentose phosphate pathway (Bettiga *et al*., 2008; Stephens *et al*., 2007), suggests that the time to coalescence for PG1 and PG3 *xylA*_2_ homologues is much shorter than the genome‐wide average. This reduction in divergence is unlikely to be caused by selectional constraints, as the *K*_a_/*K*_s_ ratio, which is an indicator of the strength and type of selection that may be acting on a gene (Li, 1993; Sharp, 1997), implies that the *xylA*_2_ gene is not experiencing a stronger level of purifying selection relative to the genome‐wide average. Importantly, these imported *xyl* genes are not part of the d‐xylose degradation operon (*xylRAFGH*), which is present in all lineages regardless of host type. The additional *xyl* genes are highly diverged from their *xylRAFGH* homologues and are therefore unlikely to have arisen via duplication. Thus, we infer that these genes have been imported via HGT from outside the *P. syringae* species complex and, although the specific function of these imported *xyl* genes is yet to be determined, we hypothesize that their presence may allow for an increase in either the rate or efficiency of d‐xylose utilization in the woody environment.

The proximity and orientation of the T3SE gene *hopV1* to the *xylRAFGH* operon suggest that *hopV1* may be co‐expressed with the inducement of the xylose operon—i.e. in the presence of d‐xylose. This mechanism may be selectively advantageous if HopV1 contributes to pathogenicity in xylose‐rich environments, such as the woody tissues of an infected woody host plant.

The alternative xylose isomerase gene (*xylA*_2_) is located next to three genes (*mocDEF*) with putative functions in the degradation of opine compounds. The *mocDEF* genes encoded by rhizobial species have been well characterized in their capacity to utilize rhizopines (Bahar *et al*., 1998), but the action of these genes is also thought to be similar to the initial stages of the degradation of aromatic hydrocarbons, such as toluene, benzene and xylene (Bahar *et al*., 2000; Suzuki *et al*., 1991). The production of opine compounds is a common feature of gall‐inducing bacterial species from the genus *Agrobacterium* (Kim and Farrand, 1996); however, the *mocDEF* genes encoded by *P. syringae* are not similar to genes in the *Agrobacterium* pathway, and there is no evidence of the remainder of this operon (*mocCABR*) in any *P. syringae* lineage. Thus, although the putative function of the *mocDEF* genes in *P. syringae* remains unclear, their presence may allow for the utilization of opine‐like molecules that are produced by other bacteria on woody plants, or as a part of an alternative and uncharacterized pathway involved in the degradation of aromatic compounds, such as toluene and xylene.

### Acquisition of the β‐ketoadipate pathway coincides with expansion into the woody niche across PGs

A number of studies have indicated the potential importance of the β‐ketoadipate operon in the ability of pathovars, such as *aesculi*, *savastanoi* and *actinidiae*, to cause disease in their respective host plants (Green *et al*., 2010; Marcelletti *et al*., 2011; Rodríguez‐Palenzuela *et al*., 2010). More recently, Bartoli *et al*. (2015) have shown a correlation between the presence of this locus and the ability of strains to grow endophytically in the stems of kiwifruit, highlighting the importance of these genes in the adaptation of *P. syringae* to that woody niche. In our extended analysis (and in agreement with the results of Bartoli *et al*., 2015), we find this operon to be present in the major expansions of *P. syringae* onto woody hosts in both PG1 and PG3. We infer this pathway to have been gained at the root of the large monophyletic cluster of woody host strains in PG3, and we hypothesize that the gain of these genes may have been the underlying factor that facilitated the remarkable diversification of this group of PG3 lineages onto a range of woody host species.

Bartoli *et al*. (2015) have suggested that the presence of the β‐ketoadipate operon in PG1 and PG3 strains is most probably the result of a single gain in the ancestor to the *P. syringae* species complex. However, our results show a reduced level of divergence between PG1 and PG3 homologues at this locus that would indicate a more recent common ancestor for these genes, relative to the genome‐wide average, and evidence for phylogenetic discordance at genes flanking the β‐ketoadipate cluster, indicative of recombination in these regions. The reduced divergence is again unlikely to be a result of selection, as the *K*_a_/*K*_s_ ratio does not indicate that these genes are experiencing unusually strong purifying selection, relative to the genome‐wide average. Thus, we suggest that the β‐ketoadipate operon was probably gained subsequent to the differentiation of PGs 1, 2 and 3 from a source most likely outside the *P. syringae* species complex, and that a recombination event between an ancestral PG1 lineage and an ancestral PG3 lineage resulted in the presence of these genes in both PGs. Given that *K*_s_ within the ‘actinidiae’ clade is about twice that of the ‘aesculi’ clade, the most likely scenario is that the operon was first acquired by a PG1 lineage, and was transferred into PG3 soon after. A number of other factors, such as the reduced divergence between the PG1 and PG3 *xylA*_2_ homologues and the phylogenetic placement of the *hopV1* gene, also point to a history of recombination between woody host lineages in PGs 1 and 3.

Although the β‐ketoadipate pathway is likely to be important for pathogenesis in pathovars such as *aesculi* and *actinidiae*, it is clearly not required for all pathogens of woody hosts. It is interesting to note the absence of this pathway from the PG1 pv. *avellanae* strains (*avel631* and *avelec1*), the causal agents of hazelnut decline. These strains are close relatives of pathovars *actinidiae*, *theae* and *morsprunorum*, and cluster as a sister clade to these pathovars. Thus, PG1 pv. *avellanae* strains, together with all PG2 pathogens of woody hosts (primarily species of fruit tree, such as cherry and apple), must use alternative metabolic pathways that are yet to be elucidated. Furthermore, it is intriguing to note the presence of these genes in the PG1 pv. *morsprunorum* str. 302280PT (*mors302280*), despite the apparent non‐pathogenicity of this strain on its plum host (Gardan *et al*., 1999; Ménard *et al*., 2003). Although further testing may be required to confirm the non‐pathogenicity of *mors302280*, we hypothesize that this strain may have lost some other component that is required for pathogenesis, either during passage or in the wild, highlighting the potential rapidity at which the transition between a pathogen and an epiphyte can occur.

### A novel approach for the detection of candidate genes from whole‐genome data

The search for associations between genotype and phenotype has been used as an analytical approach in many areas of research, particularly in relation to humans and disease (e.g. Hirschhorn and Daly, 2005). The application of the same principles to bacterial populations, however, has only recently gained traction, primarily because of the problems associated with accounting for the underlying structure of bacterial populations (e.g. Falush and Bowden, 2006). Consequently, the number of available methods for addressing these questions remains limited (but see Sheppard *et al*., 2013 for a notable alternative method). Here, we describe a novel approach for the detection of candidate genes that may be functionally involved in the expression of a given phenotype by a bacterial lineage. Our method combines phylogenetics and whole‐genome data within a statistical framework, and highlights a number of genes and associated pathways that may be involved in the adaptation of *P. syringae* to woody hosts. Further work is now required to confirm these findings, and to elucidate the potential roles of these genes in pathogenesis. Given the increasing availability of genomic data in other genera, including a number of other plant‐pathogenic microbial systems, such as *Xanthomonas* and *Phytophthora*, we suggest that our method may be useful as a first step for the rapid identification of candidate genes from whole‐genome sequence data.

## Experimental Procedures

### Strain information

We selected 26 strains of 16 different pathovars for whole‐genome shotgun sequencing. All strains have been reported to infect the woody parts of their respective host species, and to cause a range of diseases with symptoms including cankers, galls, knots and tissue necrosis. Information regarding the source, host, disease symptoms and reference is provided in Table 1 for all strains used in this study.

Freeze‐dried samples were revived by streaking onto King's B agar and incubated for 24 h at room temperature. For each strain, a single colony was selected and grown overnight in 3 mL of King's B broth for 12 h with shaking at room temperature. Laboratory passage of strains was minimized to avoid the loss of non‐essential genes, although the total length of passage since the original isolation is not known. For each isolate, cells were harvested by centrifugation of 1.5 mL of overnight culture at 1400 g for 5 min, discarding the supernatant and storing at −80°C. Genomic DNA was extracted using the DNeasy Plant Mini Kit (Qiagen, Hilden, Germany), following the standard protocol.

### Whole‐genome sequencing, assembly and annotation

For each strain, a single library with an estimated average insert of ∼270 bases was prepared by ARK Genomics (now Edinburgh Genomics, Edinburgh, UK) using Illumina Nextera reagents. Libraries were multiplexed and run on a single lane of an Illumina MiSeq benchtop sequencer by ARK Genomics, to generate datasets of 250 base paired‐end reads.

Reads containing adapter contamination were identified and trimmed using a combination of CutAdapt v1.2.1 (Martin, 2011) and TagDust v1.12 (Lassmann *et al*., 2009). Low‐quality base pairs (quality score threshold < 25) were trimmed using ConDeTri v2.2 (Smeds and Künstner, 2011). The final assembly for all strains was performed using a modified version of the SPAdes assembler v2.4.0 (Bankevich *et al*., 2012) that allowed for an increased final *k*‐mer of 229. Assembly ‘completeness’ was assessed by mapping the adapter‐ and quality‐trimmed reads to its assembly using the Bowtie2 aligner v2.2.6 (Langmead and Salzberg, 2012) and counting the proportion of data that aligned. Gene repertoire completeness was also assessed by querying a set of 40 ‘core’ bacterial proteins, recently defined by Simão *et al*. (2015), against each assembly using tblastn (*E*‐value ≤ 1e–5). All genomes were annotated with the Rapid Annotation using Subsystem Technology (RAST) online server (Aziz *et al*., 2008; Overbeek *et al*., 2013). This Whole Genome Shotgun project, including raw data, has been deposited at DDBJ/EMBL/GenBank under the BioProject accession number PRJNA287460.

### Sequence data and orthology

Genome data for an additional 38 strains were downloaded from the National Center for Biotechnology Information (NCBI) GenBank, giving a total of 64 strains of 33 pathovars. The genome sequences for certain strains, e.g. pv. *oryzae* str. 1_6, were explicitly excluded because of a high level of fragmentation, which is known to cause errors in the inference of orthology among proteins. To account for potential variation in gene content as a result of differences in annotation methodologies, all strains were re‐annotated using RAST, with the exception of the extensively curated genomes of pv. *tomato* str. DC3000, pv. *phaseolicola* str. 1448A and pv. *syringae* str. B728a.

Proteins were clustered into orthologous groups (OGs) using OrthoMCL v2.0.9 (Li *et al*., 2003; Van Dongen, 2000). The OrthoMCL pipeline first performs an all‐versus‐all blast (*E*‐value ≤ 1e–5), followed by Markov clustering (MCL), to determine clusters of orthologous proteins. MCL was performed across a range of inflation indices from 1.2 to 4.8, choosing the final value, 1.5, which maximizes the number of single‐copy OGs in all 64 strains (Swingley *et al*., 2008). The resultant list of putative OGs was subjected to a number of quality control procedures as per Nowell *et al*. (2014) to improve the inference of orthologous relationships among proteins.

### Phylogenetics and reconstruction of gene gain and loss

The evolutionary history of the core genome was estimated from the concatenated alignment of 2086 one‐to‐one (single‐copy) orthologous genes. Nucleotide alignments were generated using T‐Coffee (Notredame *et al*., 2000) and concatenated using Geneious. Gap columns were removed, giving a final alignment of 1.15 Mb in length. A maximum likelihood phylogeny was constructed in RAxML v7.2.8 (Stamatakis, 2006), using a GTR + Γ model of evolution, and 100 bootstrap resamples.

The list of OGs was converted into a binary matrix of gene occurrence and mapped onto the core genome phylogeny using GLOOME software (Cohen and Pupko, 2011; Cohen *et al*., 2008, 2010). Briefly, this method uses stochastic mapping to infer both the total number of gene gains and losses per branch and the associated probability of gain for all OGs across all branches of the phylogeny, allowing for the identification of genes with a high probability of gain (≥0.8) along specific branches of the phylogeny.

Where applicable, gained genes were functionally annotated using blast and/or blast2go (Conesa *et al*., 2005); nucleotide data for individual genes were aligned using Geneious v5.4 (Biomatters Ltd., Auckland, New Zealand) and phylogenies were constructed using PhyML v3.0 (Guindon and Gascuel, 2003; Guindon *et al*., 2010), employing the general time reversible model of evolution with four gamma‐distributed rate categories (GTR + Γ), and 100 bootstrap replicates to assess topological support.

### Distribution of T3SEs and virulence factors

Sequence data for T3SEs were downloaded from www.pseudomonas-syringae.org (16 August 2013) and combined with a multi‐species T3SE database compiled by Wang *et al*. (2012) to give a database of 1729 sequences. These were queried against the genomes using tblastn (*E*‐value ≤ 1e–5), defining presence by similarity if a hit showed a minimum of 80% identity over at least 80% query length. Putative truncation was recorded if a hit showed ≥80% identity over <80% query length. It should be noted that the ability of each putative effector to be translocated was not tested. The same schema was used for screening for a range of other virulence factors.

### Statistical modelling of correlated evolution

We modelled correlated evolution between two traits, host type and gene occurrence, using the ‘Discrete’ module of the program BayesTraits v2 (Pagel, 1994; Pagel and Meade, 2006). This method fits continuous‐time Markov models to discrete binary data, and calculates the likelihood of two hierarchically nested evolutionary models, one in which two traits are allowed to evolve independently along a phylogenetic tree and another in which the two traits evolve in a correlated (dependent) manner (Barker and Pagel, 2005; Pagel, 1994; Pagel and Meade, 2006). We define host type as a discrete binary trait designated ‘woody’ (W) or ‘herbaceous’ (H), dependent on the natural ability of an individual strain to proliferate within the woody organs of its host. Pathogenic capabilities were not tested explicitly; trait designation for host type was inferred on the basis of careful analysis of the literature for each strain. Gene occurrence was defined as a discrete binary trait, designated either ‘1’ for gene presence or ‘0’ for gene absence. Our model therefore makes two important assumptions: (i) that the host‐type trait is in fact discrete, binary and mutually exclusive—strains that may have the ability to colonize both woody and non‐woody hosts are not accounted for; and (ii) that no genes have been lost in the time between the description of each strain's pathogenicity and genome sequencing.

### Hypothesis testing and null model

The goodness of fit of the dependent versus the independent model was compared using an LR test: $\text{LR}=-2\left({\text{log}}_{e}\left(H_{0}\right)-{\text{log}}_{e}\left(H_{1}\right)\right),$ where $H_{0}$ is the likelihood of the independent model and $H_{1}$ is the likelihood of the dependent model (Pagel, 1994). A custom Perl script (available from https://github.com/reubwn/bayestraits-wrapper) was written that ran both models and calculated the LR statistic for all genes that occurred in either greater than five or fewer than 59 strains (i.e. excluding genes that were present at either a very low or very high frequency), resulting in a total of 3883 LRs.

To account for the problem of multiple testing, we constructed a null distribution of LRs that describes the random association between host preference and gene presence (Barker and Pagel, 2005). The construction of an empirically estimated null distribution negates the need for corrections, such as Bonferroni adjustment, as the null model should provide the expected distribution of LRs under the hypothesis of no association between the two traits, given a large number of individual tests. The null LR distribution was constructed by randomly permuting the gene occurrence data for each of the 3883 tested genes a total of ten times, in each case calculating a new LR statistic. The phylogeny, the H/W trait designations for each taxon and the overall proportion of gene presence relative to absence at each gene were held constant; only the occurrence profile was permuted; *P* value thresholds were then derived directly from the null distribution. An alternative null model, in which only the host‐type trait designation (H or W) was permuted, was also calculated for comparison.

## Supporting information

Additional Supporting Information may be found in the online version of this article at the publisher's website:

**Table S1** Genome assembly information.
**Table S2** Annotations for 59 genes significantly associated with the woody niche.
**Table S3** Plasmid content in genome assemblies.
**Table S4** Patterns of nucleotide divergence for selected loci.
**Table S5** Source abbreviations.
**Fig. S1** Core and pan‐genomics of the *Pseudomonas syringae* species complex.
**Fig. S2** Likelihood ratio (LR) distribution of the *Pseudomonas syringae* flexible genome.
**Fig. S3** Gene phylogeny for *hopV1*.
**Fig. S4** The xylose degradation operon in *Pseudomonas syringae*.

**Dataset S1** Sequence data for proteins inferred to have been acquired along the phylogenetic lineage leading to the *aesculi* pathovar.
