# Supplementary material for: Comparative genomics reveals genes significantly associated with woody hosts in the plant pathogen Pseudomonas syringae
Source: Mol Plant Pathol. 2016 Jul 15;17(9):1409–24. doi: 10.1111/mpp.12423 (PMC5132102; doi:10.1111/mpp.12423)
Supplement: Supplementary file 1 — Table S1 Genome assembly information. Table S2 Annotations for 59 genes significantly associated with the woody niche. Table S3 Plasmid content in genome assemblies. Table S4 Patterns of nucleotide divergence for selected loci. Table S5 Source abbreviations. Fig. S1 Core and pan‐genomics of the Pseudomonas syringae species complex. Fig. S2 Likelihood ratio (LR) distribution of the Pseudomonas syringae flexible genome. Fig. S3 Gene phylogeny for hopV1. Fig. S4 The xylose degradation operon in Pseudomonas syringae. [file MPP-17-1409-s001.docx]

# Supporting Information

Supporting Information for Nowell *et al.*, “**Comparative genomics reveals genes significantly associated with woody hosts in the plant pathogen *Pseudomonas syringae***”.

## Contents:

**Table S1** Genome assembly information.

**Table S2** Putative functions of genes associated with woody hosts.

**Table S3** Plasmid content in genome assemblies.

**Table S4** Patterns of divergence for selected loci.

**Table S5** Source abbreviations.

**Fig. S1** Core and pan genomics of the *P. syringae* species complex.

**Fig. S2** Likelihood ratio distribution of the *P. syringae* flexible genome.

**Fig. S3** Gene phylogeny for *hopV1*.

**Fig. S4** The xylose degradation operon in *P. syringae*.

**Table S1** Genome assembly information.

| Identifier^a^ | Coverage (X)^b^ | Contigs^c^ | N50 (kb) | Span (Mb) | %GC | CDS | tRNA | %Mapped^d^ |  | BUSCO^e^ |  | GenBank accession |
| --- | --- | --- | --- | --- | --- | --- | --- | --- | --- | --- | --- | --- |
| aesc2113 | 93.2 | 329 (264) | 60.9 | 6.17 | 58.3 | 5,644 | 66 | 98.4 |  | 39 |  | LIKY00000000 |
| aesc2279 | 68.4 | 322 (266) | 59.0 | 6.20 | 58.3 | 5,688 | 66 | 99.2 |  | 39 |  | LILH00000000 |
| aesc2306 | 120.4 | 290 (255) | 66.2 | 6.23 | 58.3 | 5,734 | 65 | 98.4 |  | 39 |  | LILI00000000 |
| aesc2315 | 87.9 | 288 (238) | 60.9 | 6.13 | 58.3 | 5,623 | 66 | 98.4 |  | 39 |  | LILJ00000000 |
| aesc2329 | 113.9 | 317 (263) | 60.9 | 6.24 | 58.3 | 5,797 | 65 | 99.2 |  | 39 |  | LILK00000000 |
| aesc2336 | 144.2 | 288 (248) | 66.2 | 6.21 | 58.3 | 5,717 | 66 | 98.7 |  | 39 |  | LILL00000000 |
| avii3648 | 69.3 | 323 (256) | 53.9 | 6.23 | 58.7 | 5,680 | 63 | 98.5 |  | 39 |  | LIIJ00000000 |
| brou5140 | 77.2 | 359 (302) | 43.6 | 6.12 | 57.8 | 5,784 | 63 | 99.1 |  | 39 |  | LIII00000000 |
| cast4217 | 66.3 | 219 (176) | 85.4 | 6.26 | 57.9 | 5,710 | 63 | 99.4 |  | 39 |  | LIIH00000000 |
| cera6109 | 104.4 | 352 (307) | 41.8 | 5.75 | 58.3 | 5,415 | 63 | 97.8 |  | 39 |  | LIIG00000000 |
| daph4219 | 113.1 | 369 (333) | 41.9 | 6.28 | 58.1 | 5,697 | 72 | 98.4 |  | 39 |  | LIIF00000000 |
| dend3226 | 79.7 | 219 (202) | 66.3 | 5.87 | 58.1 | 5,334 | 58 | 99.3 |  | 39 |  | LIIE00000000 |
| erio2343 | 82.7 | 128 (119) | 133.3 | 6.32 | 57.8 | 5,733 | 66 | 98.9 |  | 39 |  | LIID00000000 |
| frax5062 | 77.7 | 330 (281) | 68.9 | 6.27 | 57.9 | 5,723 | 63 | 98.6 |  | 39 |  | LIIC00000000 |
| mors2341 | 101.8 | 173 (152) | 169.2 | 6.28 | 58.1 | 5,692 | 66 | 99.6 |  | 39 |  | LIIB00000000 |
| mors5261 | 72.9 | 263 (221) | 83.1 | 6.47 | 58.4 | 5,887 | 65 | 99.3 |  | 39 |  | LIIA00000000 |
| mors5269 | 104.2 | 158 (116) | 145.9 | 6.19 | 58.1 | 5,580 | 66 | 99.2 |  | 39 |  | LIHZ00000000 |
| myri2897 | 67 | 203 (187) | 98.4 | 6.03 | 58.2 | 5,421 | 68 | 98.4 |  | 39 |  | LIHY00000000 |
| neri5067 | 54.2 | 242 (220) | 56.8 | 5.79 | 58.2 | 5,249 | 61 | 98.8 |  | 39 |  | LIHX00000000 |
| papu1754 | 41.5 | 173 (158) | 92.7 | 6.18 | 58.8 | 5,705 | 67 | 99.5 |  | 39 |  | LIHW00000000 |
| rhap4220 | 97.3 | 291 (275) | 42.7 | 5.62 | 58.3 | 5,159 | 62 | 98.4 |  | 39 |  | LIHV00000000 |
| syri2339 | 97.7 | 68 (59) | 173.3 | 6,122,834 | 59.23 | 5,246 | 67 | 99.7 |  | 39 |  | LIHU00000000 |
| syri2340 | 167.2 | 97 (82) | 246.4 | 6,182,129 | 59.13 | 5,354 | 69 | 99.1 |  | 39 |  | LIHT00000000 |
| syri7872 | 124 | 105 (73) | 212.1 | 5,900,176 | 59.34 | 5,058 | 66 | 98.8 |  | 39 |  | LIHS00000000 |
| syri7924 | 53.7 | 130 (112) | 123.5 | 6,240,460 | 59.11 | 5,478 | 66 | 99.7 |  | 39 |  | LIHR00000000 |
| ulmi1407 | 123.7 | 322 (296) | 56.9 | 6,404,243 | 57.92 | 5,933 | 66 | 98.3 |  | 39 |  | LIHQ00000000 |

^a^Unique identifier used in this study; ^b^Calculated as the total length of the trimmed reads divided by the final assembly length for each genome; ^c^Numbers in parentheses indicates the number of contigs greater than 500 bp in length; ^d^Proportion of adapter- and quality-filtered reads that map to the assembly; ^e^Number of BUSCO ‘core’ bacterial proteins, out of a total of 40, recovered from assembly (tBLASTn, *E*-value 1e–5); the only protein not found in all assemblies was COG0184, encoding a 30S ribosomal S15 protein from the Archaea. This Whole Genome Shotgun project has been deposited at DDBJ/EMBL/GenBank under the BioProject accession number PRJNA287460. The versions described in this paper are versions XXXX01000000.

**Table S2** Putative functions of genes associated with woody hosts.

| Rank | LR^a^ | Identity^b^ (%) | Coverage^c^ (%) | Description |  |
| --- | --- | --- | --- | --- | --- |
| 1 | 23.1 | 97 | 79 | Type III effector HopAY1 |  |
| 7 | 18.7 | 97 | 98 | Urease accessory protein UreG |  |
| 8 | 18.4 | 97 | 100 | CopG family transcriptional regulator |  |
| 11 | 17.7 | 100 | 100 | Urea ABC transporter permease UrtB |  |
| 13 | 17.3 | 94 | 52 | Glutamate/aspartate transport ATP-binding protein |  |
| 16 | 16.9 | 27 | 48 | Choline dehydrogenase |  |
| 17 | 16.8 | 84 | 83 | Alpha/beta hydrolase family protein |  |
| 18 | 16.8 | 100 | 100 | ATP-dependent DNA helicase RecG |  |
| 22 | 16.3 | 97 | 97 | Acetyltransferase |  |
| 23 | 16.0 | 97 | 100 | 4-oxalocrotonate tautomerase |  |
| 26 | 15.8 | 97 | 99 | Muconate cycloisomerase |  |
| 27 | 15.6 | 95 | 99 | Putative transcriptional regulator |  |
| 29 | 15.4 | 99 | 100 | Histidinol phosphatase |  |
| 31 | 15.3 | 19 | 100 | Glycoside hydrolase |  |
| 35 | 14.7 | 87 | 93 | N-acetyltransferase GCN5 |  |
| 36 | 14.6 | 99 | 100 | Urea ABC transporter substrate binding protein UrtA |  |
| 37 | 14.6 | 98 | 100 | Urea uptake/utilization operon regulator AmiR |  |
| 38 | 14.5 | 99 | 98 | Nitrogen assimilation control protein |  |
| 40 | 14.3 | 100 | 100 | HPr kinase |  |
| 42 | 14.3 | 99 | 100 | Macrolide efflux protein |  |
| 43 | 14.3 | 92 | 100 | Methyl-accepting chemotaxis protein |  |
| 44 | 14.2 | 99 | 100 | Urea ABC transporter ATPase protein UrtE |  |
| 45 | 14.2 | 92 | 99 | Type III effector HopAO1 |  |
| 48 | 14.2 | 100 | 100 | Anaerobically induced outer membrane porin OprE precursor |  |
| 49 | 14.1 | 99 | 100 | Lipid A biosynthesis, N-terminal |  |
| 50 | 14.0 | 97 | 100 | Calcium binding protein |  |
| 51 | 13.9 | 100 | 98 | AraC family transcriptional regulator |  |
| 56 | 13.2 | 62 | 97 | Sugar binding protein |  |
| 57 | 13.2 | 100 | 100 | Urea ABC transporter permease UrtB |  |

^a^Likelihood ratio score; ^b^Percent amino acid identity; ^c^Percent query coverage.

**Table S3** Plasmid content in genome assemblies.

| Strain | Number contigs^a^ | Proportion contigs (%) | Total length (bp) | Proportion genome size (%) |
| --- | --- | --- | --- | --- |
| aesc2113 | 40 | 12.1 | 113,984 | 1.8 |
| aesc2279 | 23 | 7.1 | 97,100 | 1.6 |
| aesc2306 | 42 | 14.4 | 113,341 | 1.8 |
| aesc2315 | 11 | 3.8 | 90,495 | 1.5 |
| aesc2329 | 52 | 16.3 | 153,256 | 2.4 |
| aesc2336 | 22 | 7.6 | 106,964 | 1.7 |
| avii3648 | 22 | 6.8 | 75,442 | 1.2 |
| brou5140 | 27 | 7.5 | 106,091 | 1.7 |
| cast4217 | 7 | 3.2 | 33,562 | 0.5 |
| cera6109 | 32 | 9.1 | 120,493 | 2.1 |
| daph4219 | 14 | 3.8 | 50,366 | 0.8 |
| dend3226 | 9 | 4.1 | 24,210 | 0.4 |
| erio2343 | 12 | 9.3 | 95,405 | 1.5 |
| frax5062 | 35 | 10.6 | 100,735 | 1.6 |
| mors2341 | 24 | 13.9 | 80,157 | 1.3 |
| mors5261 | 12 | 4.5 | 70,021 | 1.1 |
| mors5269 | 11 | 7.0 | 63,468 | 1.0 |
| myri2897 | 15 | 7.4 | 104,132 | 1.7 |
| neri5067 | 11 | 4.5 | 27,200 | 0.5 |
| papu1754 | 7 | 4.0 | 51,933 | 0.8 |
| phas1448A | — | — | 183,661 | 3.0 |
| rhap4220 | 12 | 4.1 | 53,962 | 1.0 |
| syri2339 | 0 | 0 | 0 | 0 |
| syri2340 | 0 | 0 | 0 | 0 |
| syri7872 | 6 | 5.7 | 2,541 | ∼0 |
| syri7924 | 0 | 0 | 0 | 0 |
| syriB728a | — | — | 0 | 0 |
| tomaDC3000 | — | — | 141,134 | 2.2 |
| ulmi1407 | 33 | 10.2 | 122,487 | 1.9 |

^a^Number of contigs putatively derived from plasmids; Pathovars with completely sequenced genomes are shown in bold for comparison; *phas1448A* and *tomaDC3000* harbour two plasmids each while *syriB728a* has none.

**Plasmid content in *P. syringae*:** Genome assemblies were queried against a database containing over 4,500 known plasmid sequences (downloaded from GenBank on June 24, 2014) using BLASTn (*E*-value ≤ 1e–5). Since contigs consisting entirely of repeat elements (such as transposons) may be derived from both plasmid and chromosomal sequences, any contig with identity (at 80% identity over 80% query length) to the three ‘finished’ (i.e., completely sequenced with no gaps) chromosomes of pv.’s *phaseolicola* str. 1448A, *syringae* str. B728a or *tomato* str. DC3000 were not included in the final estimate of plasmid length. Since this analysis excludes unsampled plasmid sequences (i.e., that were not present in the plasmid database downloaded from GenBank at the time of the analysis) and repeated sequences that may be found on both the chromosome and plasmids, the estimated proportion of plasmid sequence for our assemblies is probably an underestimate of the true value. *Pseudomonas syringae* pv. *syringae* str. B728a, in phylogroup 2, does not harbour any plasmids (Feil *et al.*, 2005). Accordingly, we find no contigs with homology to known plasmids in the three PG2 strains *syri2339*, *syri2340* and *syri7924*, while the fourth (*syri7872*) shows only a small ~2.5 kb region identified as plasmid using our criteria. However, strain *papu1754*, which also clusters within PG2, comprises at least 52 kb with homology to known plasmids.

**Feil, H., Feil, W. S., Chain, P., Larimer, F., DiBartolo, G., Copeland, A., Lykidis, A., Trong, S., Nolan, M., Goltsman, E., Thiel, J., Malfatti, S., Loper, J. E., Lapidus, A., Detter, J. C., Land, M., Richardson, P. M., Kyrpides, N. C., Ivanova, N. and Lindow, S. E.** (2005) Comparison of the complete genome sequences of *Pseudomonas syringae* pv. *syringae* B728a and pv. *tomato* DC3000. *Proc. Nat. Acad. Sci. USA*. **102**, 11064–11069.

**Table S4** Patterns of divergence for selected loci.

|  | | Distance^a^ | *K*_a_^b^ | *K*_s_ | *K*_a_/ *K*_s_ | %GC | |
| --- | --- | --- | --- | --- | --- | --- | --- |
|  |  |  |  |  |  | **PG1** | **PG3** |
| *xylA*_1_ | | 0.249 | 0.028 | 0.631 | 0.044 | 59.1 | 59.0 |
| *xylA*_2_ | | 0.068 | 0.020 | 0.118 | 0.174 | 57.7 | 58.5 |
| *β*-ketoadipate | | 0.127 | 0.007 | 0.097 | 0.067 | 61.2 | 60.9 |
|  | Within PG1 | — | 0.002 | 0.029 | 0.084 | — | — |
|  | Within PG3 | — | 0.001 | 0.007 | 0.179 | — | — |
| Core genome | | 0.215 | 0.094 | 1.644 | 0.057 | 59.9 | 59.2 |

^a^Total patristic distance (measured in substitutions per site); ^b^Values for the number of nonsynonymous substitutions per nonsynonymous site (*K*_a_) and the number of synonymous substitutions per synonymous site (*K*_s_) are given as the average of all pairwise comparisons between PG1 and PG3 homologs (except for the ‘Within PG’ estimates); *K*_a_/*K*_s_ is then the ratio of these averages. Values for the *β*-ketoadipate operon are based on a ~7.5 kb alignment of 10 genes from within the cluster. *K*_a_ and *K*_s_ were calculated using the method of Li (1993) from within the R package SeqinR (Charif and Lobry, 2007).

**Charif, D. and Lobry, J. R.** (2007) SeqinR 1.0-2: A Contributed Package to the R Project for Statistical Computing Devoted to Biological Sequences Retrieval and Analysis. In *Structural Approaches to Sequence Evolution*, Springer Berlin Heidelberg, pp. 207–232.

**Li, W.-H.** (1993) Unbiased estimation of the rates of synonymous and nonsynonymous substitution. *J. Mol. Evol.* **36**, 96–99.

**Table S5** Source abbreviations.

| Abbreviation | Source |
| --- | --- |
| ATCC | American Type Culture Collection, Virginia, USA |
| CFBP | Collection Française de Bactéries Phytopathogènes, Beaucouzé, France |
| DSM | Deutsche Sammlung von Mikroorganismen und Zellkulturen GmbH, Braunschweig, Germany |
| HRI-W | School of Life Sciences, University of Warwick, Wellesbourne, Warwick, UK |
| ICMP | International Collection of Microorganisms from Plants, Auckland, New Zealand |
| LMG | BCCM/LMG Bacteria Collection, Ghent, Belgium |
| MAFF | Ministry of Agriculture, Forestry and Fisheries, Ibaraki, Japan |
| NCPPB | National Collection of Plant Pathogenic Bacteria, York, UK |
| NRS | Forest Research, Northern Research Station, Midlothian, UK |


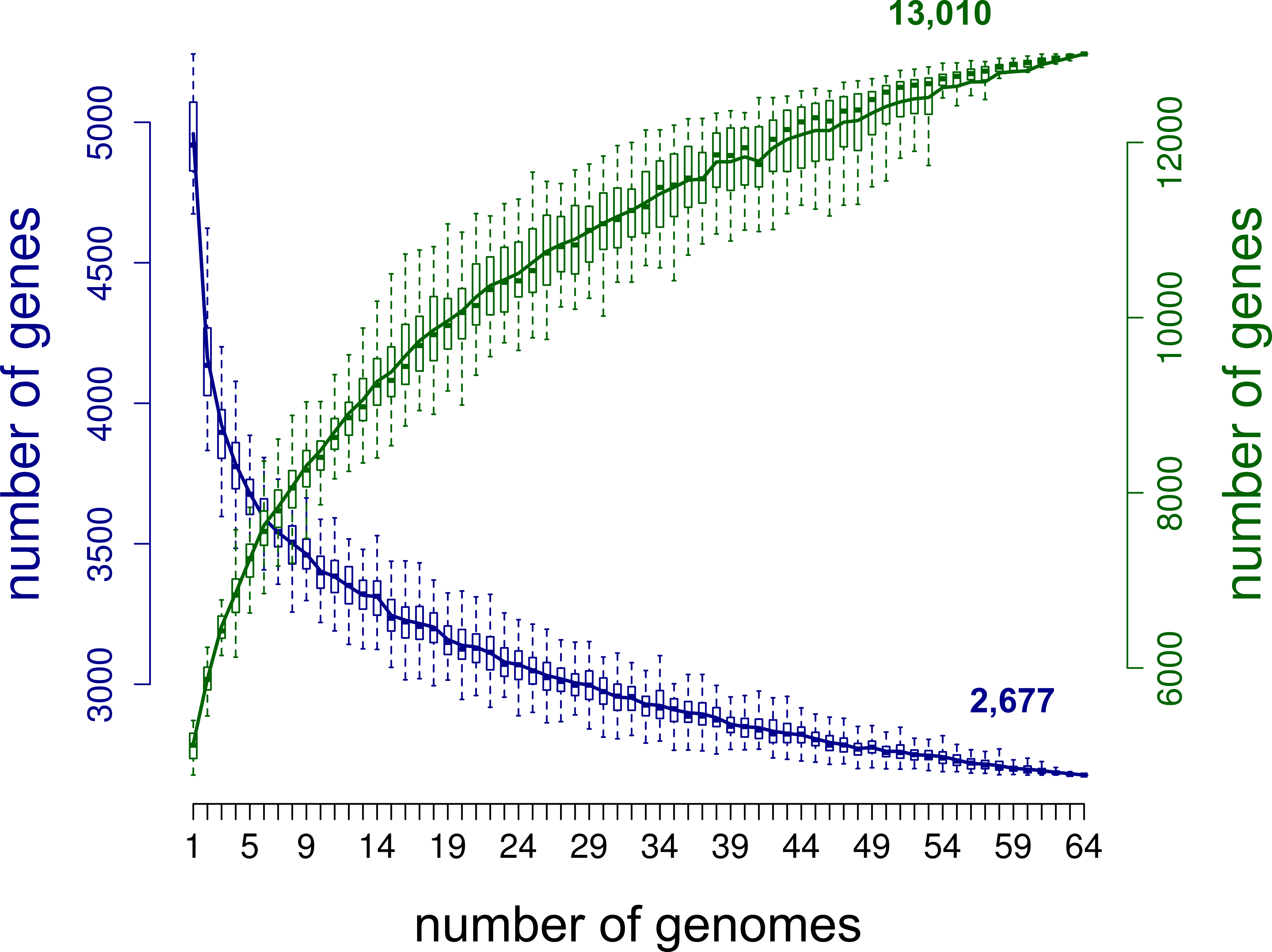


**Fig. S1** Gene accumulation curves for the core (blue scale on left-hand axis) and pan (green scale on right-hand axis) genomes of the 64 lineages of the *P. syringae* species complex used in this study. Note different scales for core and pan axes. The *x*-axis shows the number of genomes at each comparison point; box-and-whisker plots show the distribution of values for 100 random samples of genomes (with replacement).


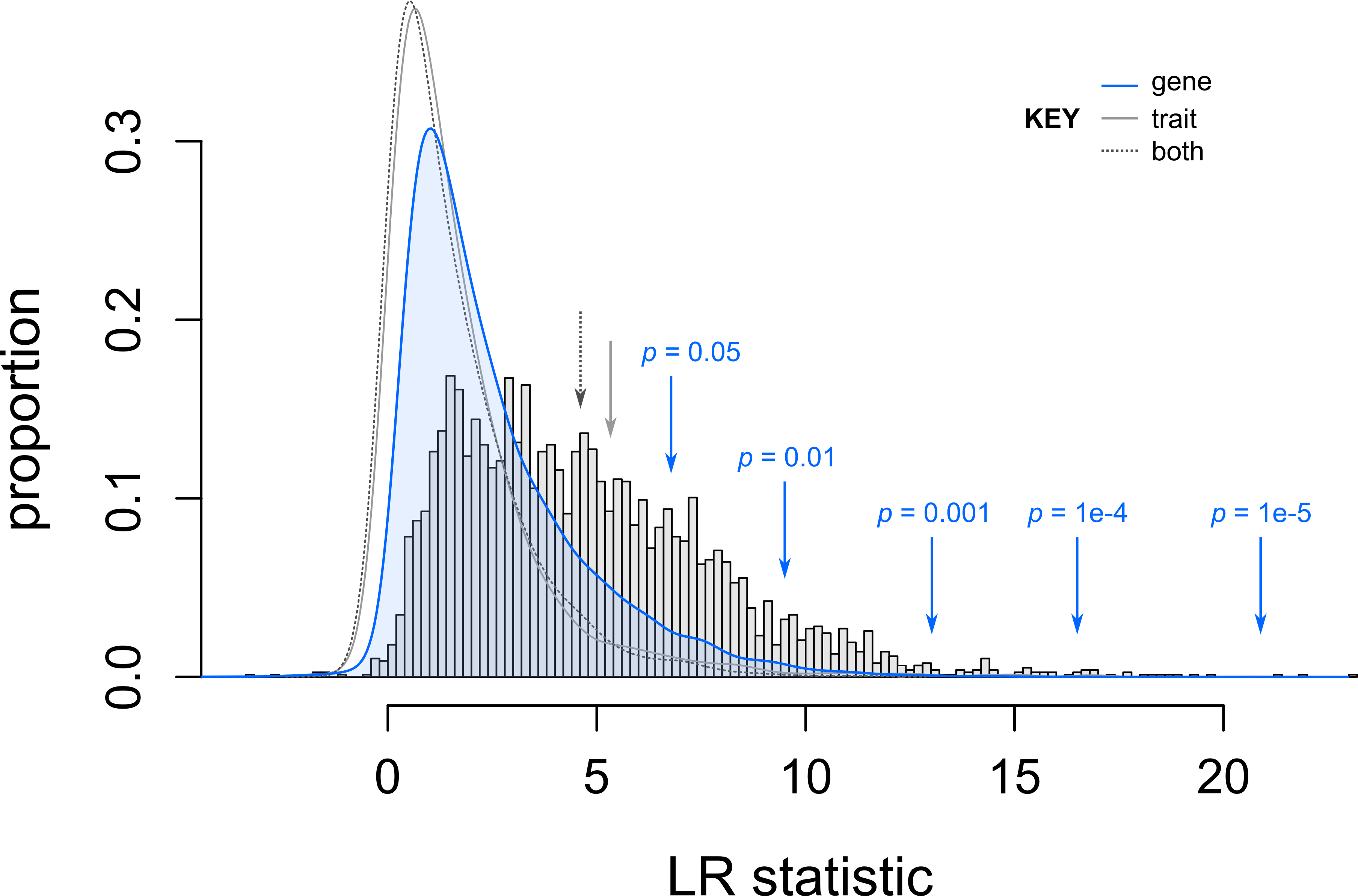


**Fig. S2** Likelihood ratio (LR) distribution for the *P. syringae* flexible genome. Gray bars show the distribution of likelihood ratio statistics for 3,883 tested sites of the *P. syringae* flexible genome measuring the support for correlated evolution between host-preference and gene presence. The estimated null based on permuting the gene occurrence data is overlaid in blue; arrows indicate *p*-value thresholds based on this null. Also shown are two alternative null models, where either the traits data (solid light gray) or both trait data and gene occurrence (dashed dark gray) have been randomised; the 5% *p*-value thresholds are also indicated with arrows for these alternative null distributions, for comparison.

**Note on null model selection:** The number of genes inferred to be significantly associated with the woody niche under the different null models is indicated below. We observed that the null distribution in which gene occurrence only was permuted (blue line in **Fig. S2** above) was the most conservative at the 5% false positive threshold; thus we compare our data to these *p*-value thresholds in subsequent analyses.

| Null | LR threshold at *p* ≤ 0.05 | Expected Type I at *p* ≤ 0.05 | Observed genes with LR ≥ 0.05 threshold | Inferred number of associated genes at *p* ≤ 0.05 |
| --- | --- | --- | --- | --- |
| Gene | 6.78 | 194 | 899 | 705 |
| Trait | 5.33 | 195 | 1,426 | 1,231 |
| Both | 4.61 | 195 | 1,757 | 1,562 |


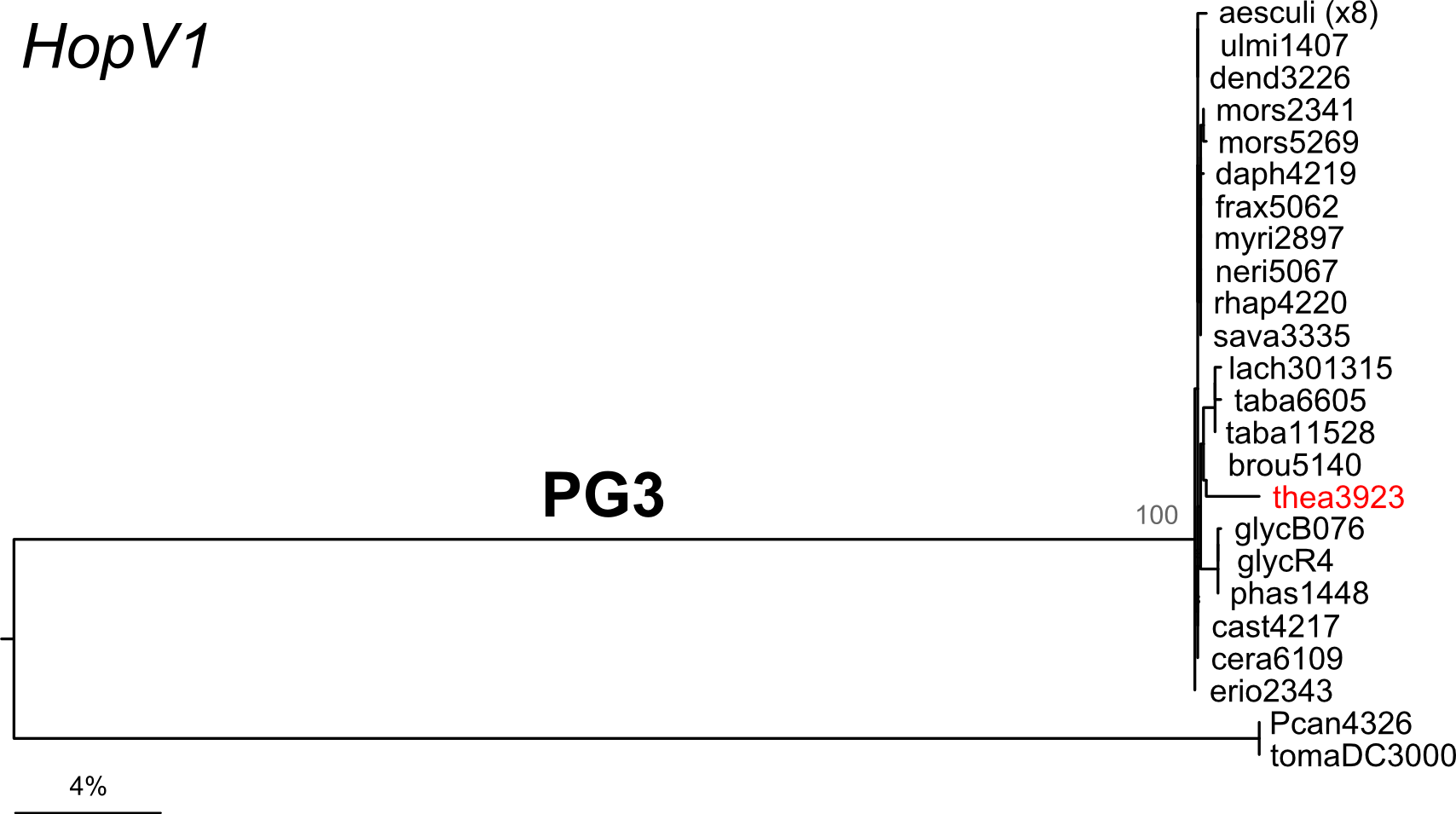


**Fig. S3** Gene phylogeny for *hopV1*. The PG1 strains *thea3923* is highlighted in red. Grey numbers indicate bootstrap support; tree is midpoint rooted; scale bar indicates 0.04 nucleotide substitutions per site.


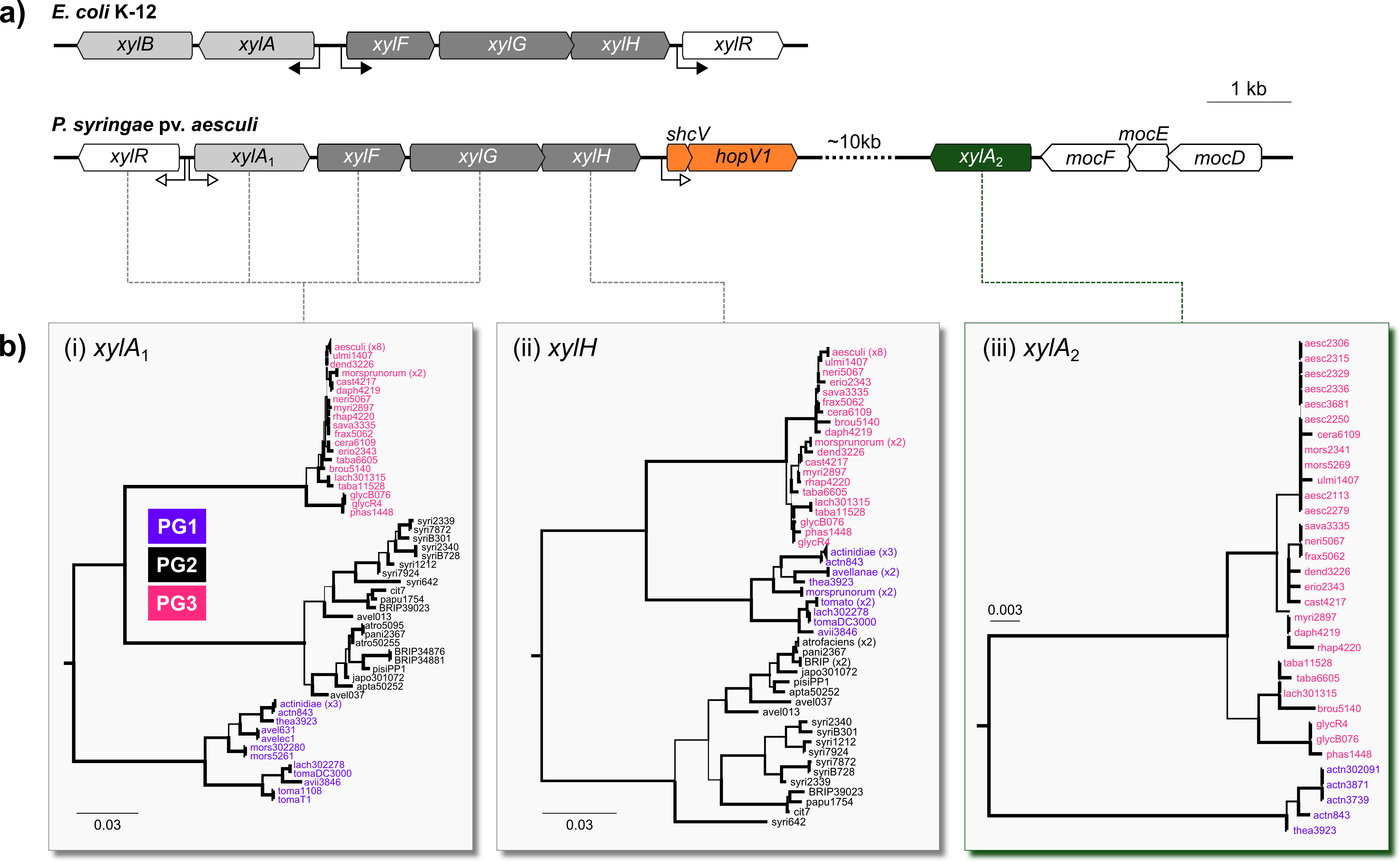


**Fig. S4** Structure and evolution of the xylose degradation operon in *P. syringae*. **(a)** Comparison with *Escherichia coli* K-12 (Song and Park, 1997) shows multiple gene rearrangements within this region, including the loss of the xylulokinase gene (*xylB*), which is located elsewhere in the *P. syringae* genome, and the rearrangement of *xylAFGH* into a single transcriptional unit. Light grey indicates metabolic enzymes, dark grey indicates transport components, the regulatory gene *xylR* is shown in white. The position of the T3SE gene *hopV1*, immediately downstream of the *xylRAFGH* operon, is shown in orange. Confirmed and predicted promoter regions are indicated by filled and open arrows respectively. **(b) (i)** Individual gene phylogenies for *xylR*, *xylA*_1_, *xylF* and *xylG* are similar to that of the core genome (only *xylA*_1_ is shown). **(ii)** Phylogeny for *xylH* is incongruent with that of the core genome. **(iii)** Gene phylogeny for the alternative xylose isomerase (*xylA*_2_). Taxa are coloured blue, black and pink corresponding to PG1, PG2 and PG3 respectively. Branch thicknesses are drawn relative to bootstrap support (thicker indicates higher support; no minimum bootstrap threshold). Scale bars represent 0.03 subs/site for (i) and (ii), and 0.003 subs/site for (iii). Trees are rooted with *Pcan4326* (not shown) for (i) and (ii), whereas (iii) is midpoint rooted.

**Song, S. and Park, C.** (1997) Organization and regulation of the D-xylose operons in *Escherichia coli* K-12: XylR acts as a transcriptional activator. *J. Bacteriol.* **179**, 7025–7032.
